# A Prognostic Model of 15 Immune-Related Gene Pairs Associated With Tumor Mutation Burden for Hepatocellular Carcinoma

**DOI:** 10.3389/fmolb.2020.581354

**Published:** 2020-11-13

**Authors:** Junyu Huo, Liqun Wu, Yunjin Zang

**Affiliations:** Liver Disease Center, The Affiliated Hospital of Qingdao University, Qingdao, China

**Keywords:** hepatocellular carcinoma, tumor mutation burden, immune, prognostic, signature

## Abstract

**Introduction:**

Tumor mutation burden (TMB) is an emerging biomarker for immunotherapy of hepatocellular carcinoma (HCC), but its value for clinical application has not been fully revealed.

**Materials and Methods:**

We used the Wilcox test to identify the differentially expressed immune-related genes (DEIRGs) in groups with high and low TMB as well as screened the immune gene pairs related to prognosis using univariate Cox regression analysis. A LASSO Cox regression prognostic model was developed by combining The Cancer Genome Atlas Liver Hepatocellular Carcinoma (TCGA-LIHC) with the International Cancer Genome Consortium (ICGC) LIRI-JP cohort, and internal (TCGA, ICGC) and external (GSE14520) validation analyses were conducted on the predictive value of the model. We also explored the relationship between the prognostic model and tumor microenvironment via the ESTIMATE algorithm and performed clinical correlation analysis by the chi-square test, revealing its underlying molecular mechanism with the help of Gene Set Enrichment Analysis (GSEA).

**Results:**

The prognostic model consisting of 15 immune gene pairs showed high predictive value for short- and long-term survival of HCC in three independent cohorts. Based on univariate multivariate Cox regression analysis, the prognostic model could be used to independently predict the prognosis in each independent cohort. The immune score, stromal score, and estimated score values were lower in the high-risk group than in the low-risk group. As shown by the chi-square test, the prognostic model exhibited an obvious correlation with the tumor stage [American Joint Committee on Cancer tumor–node–metastasis (AJCC-TNM) (*p* < 0.001), Barcelona Clinic Liver Cancer (BCLC) (*p* = 0.003)], histopathological grade (*p* = 0.033), vascular invasion (*p* = 0.009), maximum tumor diameter (*p* = 0.013), and background of liver cirrhosis (*p* < 0.001). GSEA revealed that the high-risk group had an enrichment of many oncology features, including the cell cycle, mismatch repair, DNA replication, RNA degradation, etc.

**Conclusion:**

Our research developed and validated a reliable prognostic model associated with TMB for HCC, which may help to further enrich the therapeutic targets of HCC.

## Introduction

Hepatocellular carcinoma (HCC) is the most frequent primary liver malignancy (more than 90%) and is often accompanied by chronic hepatitis or cirrhosis (Kanwal and Singal, 2019). Because of the hidden and rapid progress of HCC, most of the patients were diagnosed in the middle and advanced stages, and a considerable proportion of early HCC patients who received surgical treatment experienced relapse (Imamura et al., 2003), so the prognosis remains very poor.

Recently, as science and technology have developed continuously, the treatment of HCC is also constantly updated. In addition to traditional hepatectomy, chemotherapy, radiotherapy, liver transplantation, transcatheter arterial chemoembolization, ablation and other treatments, targeted therapies such as sorafenib, lenvatinib, and regorafenib are also gradually applied in clinical practice, but there is still a lack of effective methods for the treatment of advanced HCC (Couri and Pillai, 2019). Immunotherapy is a new way to treat HCC (Floudas et al., 2019), in which immunosuppressive checkpoint inhibitors have become a potential effective treatment for patients with advanced HCC (El Dika et al., 2019).

Tumor mutation burden (TMB) is defined as the total replacement and insertion/deletion mutation number for each megabase in the exon coding region regarding the evaluated gene of a tumor sample (Galuppini et al., 2019). It is a new biomarker for predicting the benefit of the treatment of tumor immune checkpoint inhibitors (ICIs) for various kinds of tumors (Goodman et al., 2017), such as lung cancer, colorectal cancer, prostate cancer, and breast cancer (Antonarakis, 2019; Schrock et al., 2019; Alborelli et al., 2020; Jang et al., 2020). Although increasing evidence has shown that the higher the TMB is, the more new antigens can be recognized by T cells and the better the effect of immunotherapy is (Kim et al., 2019), research on the interaction between TMB and HCC prognosis is still relatively insufficient.

This study focused on exploring the prognostic value of the TMB for HCC and identified immune-related genes correlated with TMB levels. A prognostic model of 15 immune-related gene pairs associated with tumor mutation burden for HCC was developed and validated in this research. In addition, we found that the prognosis model was related to the tumor microenvironment through the ESTIMATE algorithm, which is of great significance for precisely treating HCC.

## Materials and Methods

### Data Collection

**FIGURE 1:**
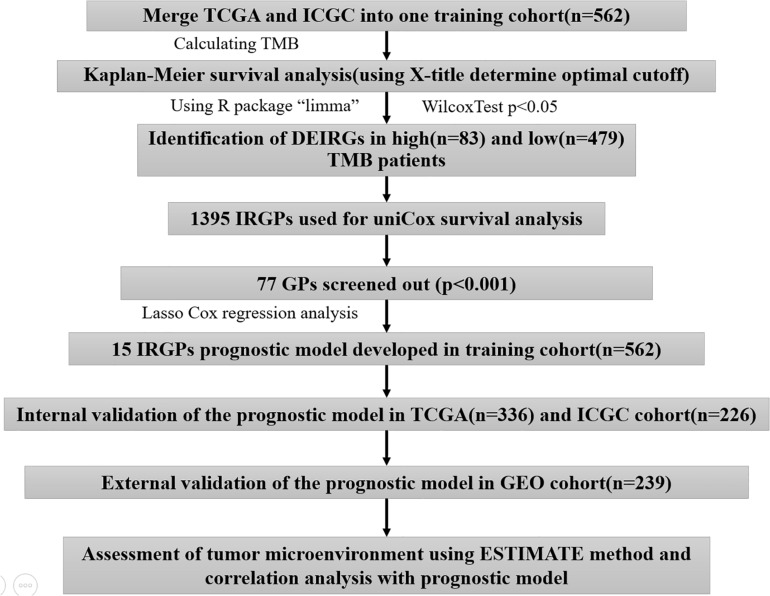
The workflow chart of this study.

A total of 801 HCC patients with complete prognostic information from three independent datasets were included in this study, and the number of cases in each independent dataset was more than 200. We obtained the RNA sequencing and gene mutation profile and clinical information from The Cancer Genome Atlas Liver Hepatocellular Carcinoma (TCGA-LIHC)^1^ and International Cancer Genome Consortium (ICGC), LIRI-JP)^2^, datasets, respectively. The gene expression files of the two datasets were all based on the Illumina HiSeq RNA Seq platform, and we merged them into one cohort for subsequent analyses. The gene expression files and corresponding clinical data were downloaded from the Gene Expression Omnibus (GEO-GSE14520) database^3^ for external validation. To exclude other factors influencing patient survival, our study did not include patients with a survival time of <1 month. The clinical information of HCC patients is shown in Supplementary Table S1. The acquisition of the above data fully complied with the access regulations and use principles of the above database. Figure 1 displays the workflow.

### Immune Gene Identification Related to TMB

We calculated the TMB with a Perl script based on the somatic mutation file of the TCGA-ICGC cohort (Zhang et al., 2019). The gene mutation waterfall map generated with the R package “GenVisR” and the optimal cutoff derived from X-title analysis were investigated to determine the association of the TMB with survival outcomes (Zhang et al., 2013; Ye et al., 2017). The cutoff value was taken into account to divide patients into high- and low-TMB groups. Then, we estimated the fraction of the 22 subtype immune cells in every sample through the CIBERSORT algorithm. The CIBERSORT algorithm has the function of estimating complicated tissue cell composition according to standardized gene expression data (Chen et al., 2018). The results of flow cytometry showed that CIBERSORT can accurately predict the composition of immune cells in HCC tissues (Newman et al., 2015). The Wilcoxon rank-sum test assisted in comparing the difference between the two groups with regard to the immune infiltrate abundance by virtue of the R package “vioplot.” A gene list related to immunity came from the Immunology Database and Analysis Portal (ImmPort)^4^, which provides essential immunology data for studying cancer (Dunn et al., 2015). We used the Wilcoxon test in the R package “limma” to screen the differentially expressed immune genes (DEIRGs) between the two groups with the criteria of FDR < 0.05. The R package “clusterProfiler” was used to annotate the biological process in which DEIRGs participate.

### Construction and Validation of the LASSO Cox Regression Immune Gene Pair Prognostic Model

We paired the DEIRGs. In each immune gene pair (IGP), when the former gene exhibited a higher expression relative to the latter gene, the value was assigned to 1. In contrast, if the expression level of the former gene was lower than that of the latter gene, the value was assigned to 0, and immune gene pairs with a proportion of “0” or “1” <20% were excluded. This approach was a major advantage in our study because the IGPs were generated by a pairwise comparison and were entirely based on the gene expression in the same patient, which can overcome the batch effects of the different platforms and does not require the data to be scaled and normalized. A total of 1,395 IGPs were obtained after screening. Univariate Cox regression analysis was performed to identify the immune gene pairs associated with prognosis (*p* < 0.001). Prognostic-associated IGPs obtained from the univariate Cox regression analysis assisted in constructing the least absolute shrinkage and selection operator (LASSO) Cox regression model. The LASSO algorithm with penalty parameter tuning performed via a 10-fold cross-validation was applied to exclude IGPs that may be highly correlated with other IGPs. A subset of IGPs was determined by shrinking the regression coefficient using a penalty proportional to their size. The IGPs with non-zero regression coefficients were retained for subsequent multivariate Cox regression analyses. A risk score model was constructed using the value of these prognostic IGPs with the regression coefficient (β) from the multivariate Cox proportional hazards regression analysis. The “glmnet” R package was used for LASSO regression analysis of prognostic IGPs. The median risk score was used to classify patients into groups with high and low risks. The R package “survminer” and “survivalROC” helped to generate the Kaplan–Meier survival and ROC curves of the risk score for estimating the predictive power featured by the model. The log-rank test was conducted to compare the two groups in terms of the survival curve, and *p* < 0.05 was regarded as statistically significant. We divided the training cohort into TCGA and ICGC cohorts for the internal validation, and the external validation was conducted by using the GSE14520 cohort.

### Independence Validation of the Prognostic Model

Univariate and multivariate Cox regression analysis was employed to examine if the risk score was an independent predictor of HCC prognosis in each independent cohort (TCGA, ICGC, and GSE14520).

### Correlation Analysis Between the Prognostic Model and Clinical Features

We performed a chi-square test to analyze the clinical characteristics of the two groups to explore the reasons for the differences in prognosis. A *p* < 0.05 demonstrated statistical significance.

### Correlation Analysis Between Prognostic Model and Tumor Microenvironment

The ESTIMATE algorithm, a tool to predict the purity of tumors, was employed for calculating the microenvironment scores of tumors (including stromal, immune, and estimate scores), and gene expression data were adopted for calculating infiltrating stromal cells or immune cells in the tumor tissues (Charoentong et al., 2017). We divided patients into high- and low-risk groups considering the median value possessed by three kinds of scores and conducted Kaplan–Meier survival analysis to explore their impact on the prognosis of HCC. We also used the Wilcoxon test to compare the differences between the three kinds of scores in the two groups, using the Pearson correlation test to analyze the association of risk score with the three scores (including the stromal, immune, and estimate scores).

### Gene Set Enrichment Analysis

Gene set enrichment analysis was conducted in the two groups from three independent cohorts to explore potential molecular mechanisms (Shi and Walker, 2007), and cp.kegg.v7.1.symbols.gmt served as a reference gene set, with a nominal *p* < 0.05 as the threshold to screen the significant enrichment pathway in different risk groups.

## Results

### Prognostic Significance of Tumor Mutation Burden in HCC

**FIGURE 2:**
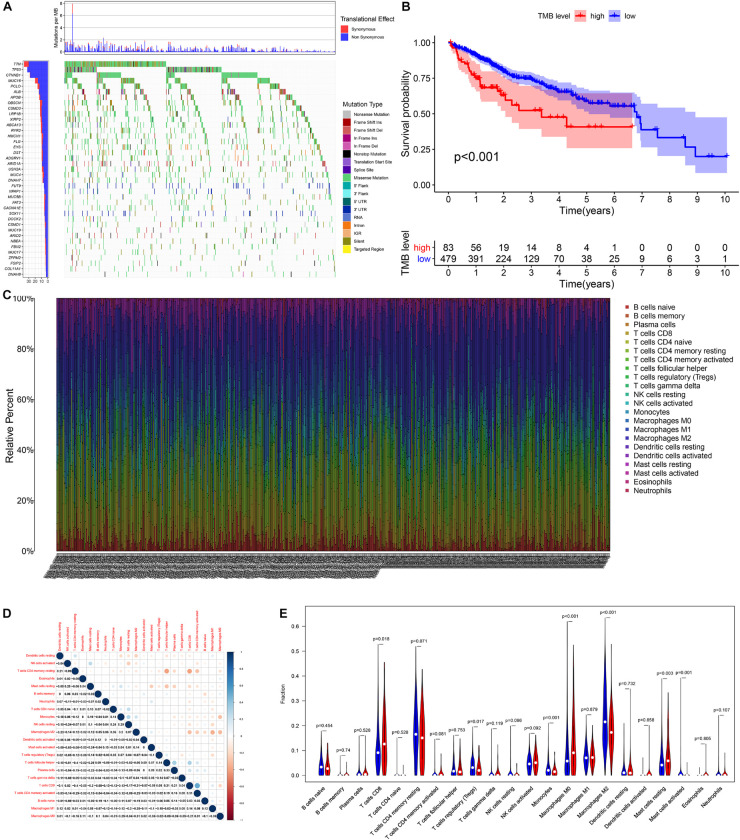
The relationship among tumor mutation burden, immune infiltration, and prognosis of HCC. **(A)** The waterfall plot mutation profiles in HCC samples from the combined TCGA and ICGC datasets. **(B)** The Kaplan–Meier survival curve regarding the TMB and overall survival. **(C)** The bar plot of 22 specific immune fractions represented by various colors in each sample are shown in the bar plot. **(D)** Correlation matrix of all 22 immune cell proportions. **(E)** The violin plot of different infiltration levels of immune cells between high- and low-TMB patients.

According to the analysis results of X-title software, TMB = 4.2 was used to divide patients into high- and low-TMB groups and had the greatest impact on the overall survival (OS) of patients with HCC (Figures 2A,B). By observing the survival curve, we can observe that the high-TMB group exhibited an obviously lower OS than the low-TMB group (log-rank *p* < 0.001).

### Relationship Between TMB and Immune Infiltration

We found that the infiltration levels of various immune cells were different between the high- and low-TMB groups. For example, high-TMB group had an obviously higher infiltration level of CD8 T cells, M0 macrophages, and mast cells than the low-TMB group, while the infiltration proportion of monocytes, regulatory T cells (Tregs), M2 macrophages, and mast cells activated in the low-TMB group was relatively high (Figures 2C–E). This indicates that the tumor immune microenvironment (TIME) is closely related to TMB. These results also provide insights for our subsequent research.

### Identification of Differentially Expressed Immune-Related Genes

**FIGURE 3:**
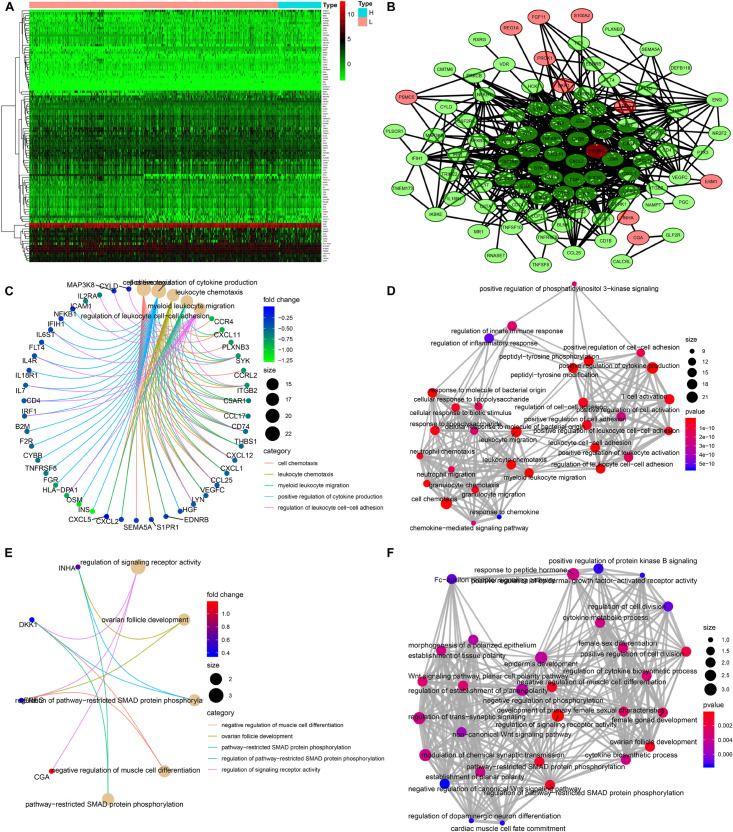
Identification of differentially expressed immune-related genes (DEIRGs) between high- and low-TMB patients. **(A)** Heatmap of DEIRGs between high- and low-TMB patients. **(B)** Protein–protein interaction plot of DEIRGs between high- and low-TMB patients (green represents upregulated genes in the low-TMB group, and red represents upregulated genes in the high-TMB group). **(C,D)** GO enrichment analysis of DEIRGs. **(E,F)** KEGG enrichment analysis of TRMGs.

A total of 103 immune-related genes with significant differences in expression between the high- and low-TMB groups were screened; of these genes, 89 and 14 were upregulated in the low- and high-TMB groups, respectively (Figures 3A,B). The genes significantly enriched in the low-TMB group could positively regulate cytokine production, leukocyte cell–cell adhesion, leukocyte chemotaxis, etc. (Figures 3C,D). The genes significantly enriched in the high-TMB group could regulate signaling receptor activity, phosphorylation, pathway-restricted SMAD protein phosphorylation, etc. (Figures 3E,F).

### LASSO Cox Regression IGP Prognostic Model Construction

**FIGURE 4:**
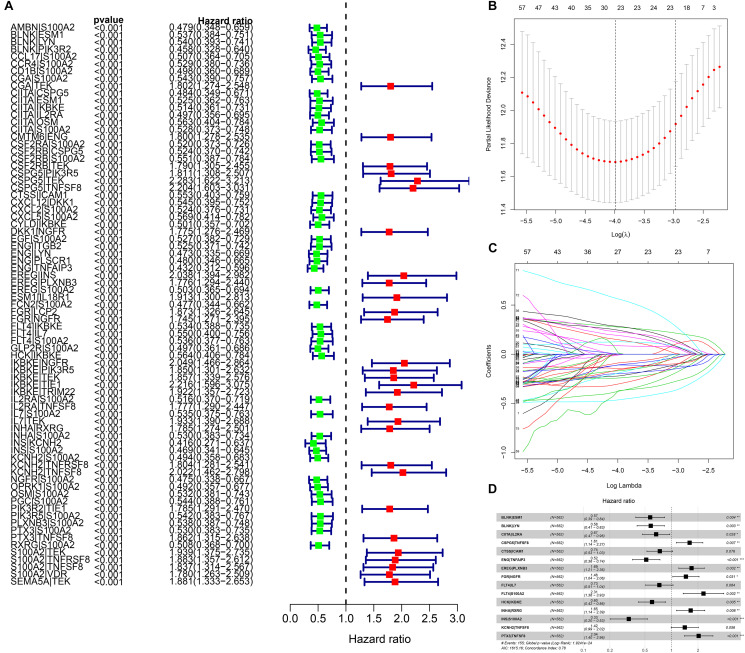
The building process of the prognostic signature. **(A)** Forrest plot of prognostic DEIRGs identified by univariate Cox and Kaplan–Meier survival analyses. **(B–D)** The establishment of the prognostic model based on LASSO penalized Cox analysis.

**FIGURE 5:**
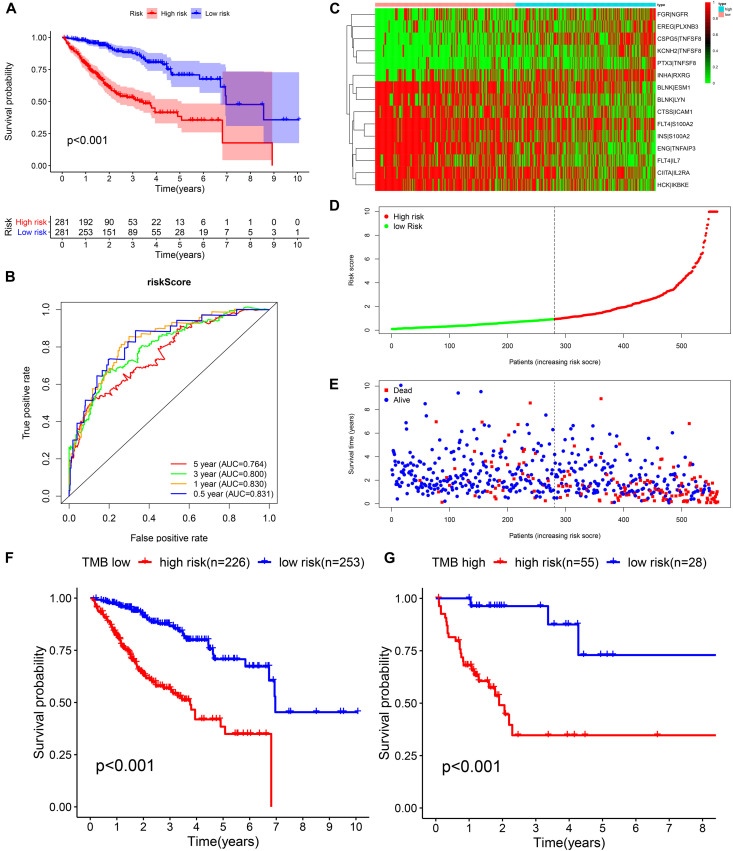
Construction of the prognostic model in the training cohort. **(A,B)** Kaplan–Meier survival analysis and time-dependent ROC analysis for predicting the overall survival of patients in the training cohort using the risk score. **(C–E)** Heatmap of the 15 gene pairs, the distribution of the risk score, and the survival status of patients. **(F)** Kaplan–Meier survival analysis for predicting the overall survival of patients in the low-TMB group using the risk score. **(G)** Kaplan–Meier survival analysis of predicting the overall survival of patients in the high-TMB group using the risk score.

We identified 77 gene pairs that exhibited an obvious relation to OS through univariate Cox regression analysis (Figure 4A), of which 29 were prognostic risk factors (HR > 1) and 48 were prognostic protective factors (HR < 1). After LASSO regression and multivariate Cox regression analyses, 15 immune gene pairs were included in our prognostic model (Figures 4B–D). We calculated each patient’s risk score following the formula (the list of gene pairs and calculation coefficients are shown in (Table 1) and divided 562 patients into high- and low-risk groups by considering the median value (0.9429) of the risk score. As shown by the survival curve, the OS of the high-risk group was obviously lower than that of the low-risk group (*p* < 0.001) (Figure 5A). The areas under curve (AUCs) for the risk score predicting overall survival at 0.5, 1, 3, and 5 years were 0.831, 0.830, 0.800, and 0.764, respectively (Figure 5B). The number of deaths increased with the increase of risk score (Figures 5C–E), and regardless of the status of tumor mutation burden, the high-risk group showed poor prognosis (Figures 5F,G). These results preliminarily indicated that it is effective to stratify the prognosis of patients according to the risk score.

**TABLE 1:** The list and coef of IGPs.

IGPs	Coef
BLNK| ESM1	−0.556894158
BLNK| LYN	−0.541824561
CIITA| IL2RA	−0.401209687
CSPG5| TNFSF8	0.474599318
CTSS| ICAM1	−0.305089132
ENG| TNFAIP3	−0.660506625
EREG| PLXNB3	0.525553186
FGR| NGFR	0.377839595
FLT4| IL7	−0.317118995
FLT4| S100A2	0.836862911
HCK| IKBKE	−0.50853469
INHA| RXRG	0.50223053
INS| S100A2	−1.116354541
KCNH2| TNFSF8	0.347981123
PTX3| TNFSF8	0.710891356

### Validation of the Prognostic Model in the TCGA and ICGC Cohorts

**FIGURE 6:**
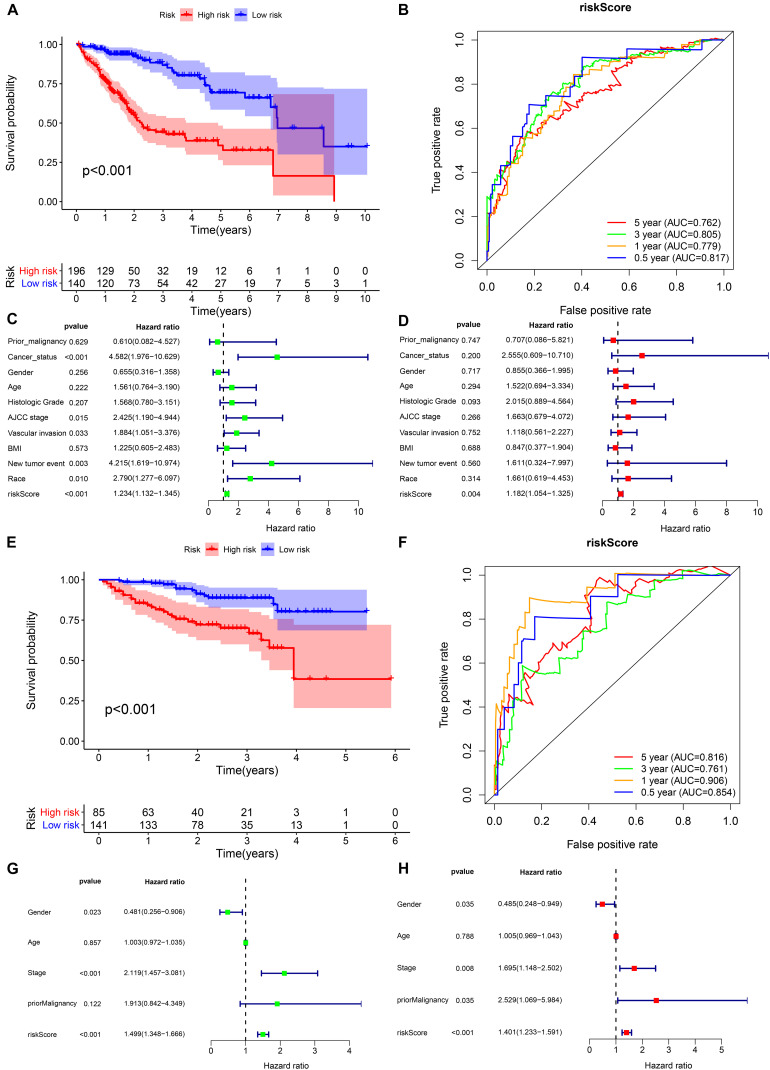
Internal validation of the prognostic model in the TCGA and ICGC cohorts. **(A,B)** Kaplan–Meier survival analysis and time-dependent ROC analysis of predicting the overall survival of patients in the TCGA cohort using the risk score. **(C,D)** Univariate and multivariate regression analyses of the relation between the risk score and clinicopathological characteristics regarding overall survival in the TCGA cohort (green represents univariate analysis, and red represents multivariate analysis). **(E,F)** Kaplan–Meier survival analysis and time-dependent ROC analysis for predicting the overall survival of patients in the ICGC cohort using the risk score. **(G,H)** Univariate and multivariate regression analyses of the relation between the risk score and clinicopathological characteristics regarding the overall survival in the ICGC cohort (green represents univariate analysis, and red represents multivariate analysis).

In the TCGA cohort, the prognosis of the high-risk group was significantly worse (Figure 6A). The AUCs for the risk score predicting overall survival at 0.5, 1, 3, and 5 years were 0.817, 0.779, 0.805, and 0.762, respectively (Figure 6B). As shown in the univariate multivariate Cox regression analysis, the risk score could serve to independently predict prognosis (Figures 6C,D). In accordance with the TCGA cohort results, the high-risk group also showed a lower overall survival rate in the ICGC cohort (Figure 6E). The AUCs for risk score predicting overall survival at 0.5, 1, 3, and 5 years were 0.854, 0.906, 0.761, and 0.816, respectively (Figure 6F). Similarly, the risk score was one of the prognostic indicators independent of other clinical factors in this cohort (Figures 6G,H). The results of internal validation demonstrated that the risk score has a high accuracy for predicting HCC prognosis.

### External Validation of the Prognostic Model in the GEO Cohort

**FIGURE 7:**
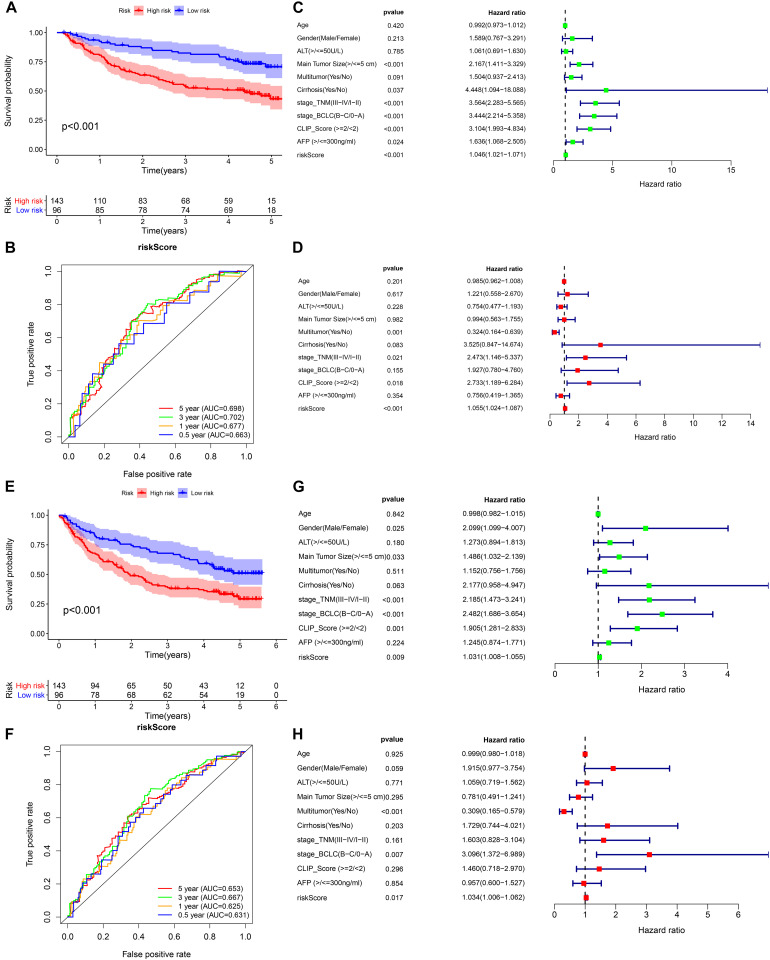
External validation of the prognostic model in the GSE14520 cohort. **(A,B)** Kaplan–Meier survival analysis and time-dependent ROC analysis for predicting the overall survival of patients in the GSE14520 cohort using the risk score. **(C,D)** Univariate and multivariate regression analyses of the relation between the risk score and clinicopathological characteristics regarding the overall survival in the GSE14520 cohort (green represents univariate analysis, and red represents multivariate analysis). **(E,F)** Kaplan–Meier survival analysis and time-dependent ROC analysis for predicting the recurrence-free survival of patients in the GSE14520 cohort using the risk score. **(G,H)** Univariate and multivariate regression analyses of the relation between the risk score and clinicopathological characteristics regarding the recurrence-free survival in the GSE14520 cohort (green represents univariate analysis, and red represents multivariate analysis).

We selected GSE14520 from the GEO database as an external dataset to validate the prognostic model because of its large sample size (*n* = 239) and complete clinical data. The risk score regarding each patient in the cohort was calculated based on the previous formula, and patients were assigned into either high- or low-risk group by taking into account the unified cutoff value (0.9429). Consistent with previous studies, the high-risk group had remarkably lower OS and recurrence-free survival rates than the low-risk group (Figures 7A,E). The AUCs for the risk score predicting overall survival at 0.5, 1, 3, and 5 years were 0.663, 0.677, 0.702, and 0.698, respectively (Figure 7B). The AUCs for the risk score predicting recurrence-free survival (RFS) at 0.5, 1, 3, and 5 years were 0.631, 0.625, 0.667, and 0.653, respectively (Figure 7F). As revealed by univariate multivariate Cox regression analysis, the risk score could be used to independently predict OS and RFS (Figures 7C,D,G,H). The results of the external validation proved that the prognostic model we constructed had general applicability and high stability in predicting the prognosis of HCC.

### Prognostic Assessment of the Prognostic Model in the Whole Cohort

**FIGURE 8:**
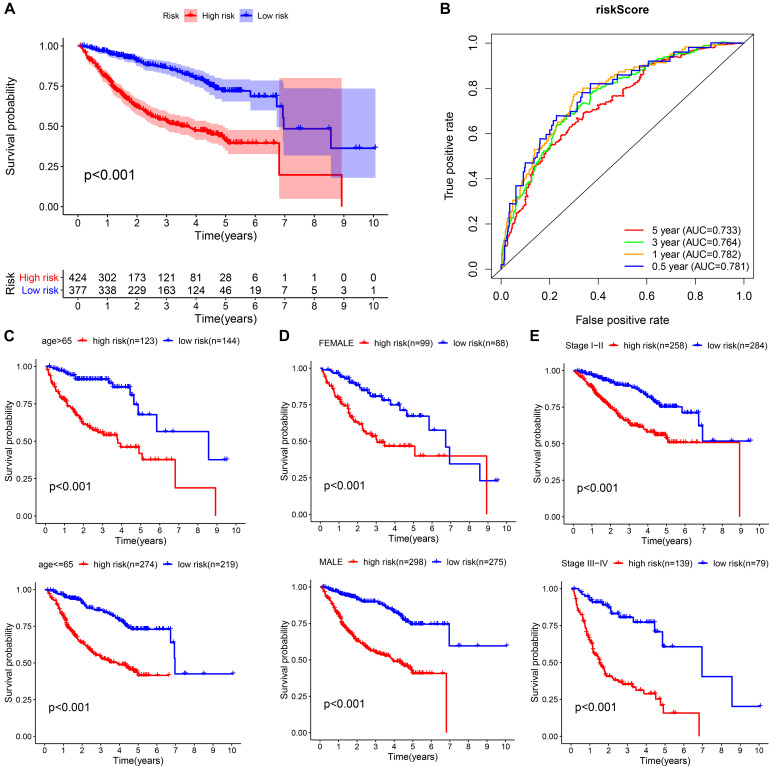
Validation of the universal applicability of the prognostic model in the whole cohort. **(A,B)** Kaplan–Meier survival analysis and time-dependent ROC analysis for predicting the overall survival of patients in the whole cohort using the risk score. **(C–E)** Subgroup Kaplan–Meier survival analysis according to different clinical features.

We integrated all the research objects into one whole cohort (*n* = 801) for analysis, and as expected, the high-risk group presented a significantly poor prognosis (Figure 8A). The AUCs for risk score predicting overall survival at 0.5, 1, 3, and 5 years were 0.781, 0.782, 0.764, and 0.733, respectively, in whole cohort (Figure 8B). For survival analysis, we divided all subjects into six subgroups according to their common clinical characteristics (age, sex, and stage). In each subgroup, the high-risk group showed an obviously lower OS than the low-risk group (Figures 8C–E). These results further confirmed that the risk score is a reliable tool for the prognostic evaluation of HCC.

### Clinical Correlation Analysis of the Prognostic Model

The chi-square test demonstrated that the prognosis model was significantly correlated with tumor stage [AJCC-TNM (*p* < 0.001), BCLC (*p* = 0.003)], histopathological grade (*p* = 0.033), vascular invasion (*p* = 0.009), maximum tumor diameter (*p* = 0.013), and background of liver cirrhosis (*p* < 0.001) (Tables 2–4).

**TABLE 2:** The chi-square test of the relation between risk score and clinical features in TCGA cohort.

Clinical feature	Risk score		c2	*p*
	High risk n(%)	Low risk n(%)		
Age			0.037	0.848
>65	72(59.02%)	50(40.98%)		
≤65	124(57.94%)	90(42.06%)		
Gender			3.244	0.072
MALE	126(55.02%)	103(44.98%)		
FEMALE	70(65.42%)	37(34.58%)		
Histologic_grade			4.543	0.033
G1-2	112(53.59%)	97(46.41%)		
G3-4	80(65.57%)	42(34.43%)		
New_tumor_event_after_initial_treatment			0.643	0.423
YES	98(59.76%)	66(40.24%)		
NO	88(55.35%)	71(44.65%)		
Pathologic_stage			3.703	0.054
Stage I–II	129(54.89%)	106(45.11%)		
Stage III–IV	55(67.07%)	27(32.93%)		
Person_neoplasm_cancer_status			2.846	0.092
WITH TUMOR	91(62.76%)	54(37.24%)		
TUMOR FREE	94(53.41%)	82(46.59%)		
BMI			0.566	0.452
>25	82(55.03%)	67(44.97%)		
≤25	96(59.26%)	66(40.74%)		
Race			1.861	0.172
White	89(54.27%)	75(45.73%)		
Asian and others	100(61.73%)	62(38.27%)		
Vascular tumor cell type			6.887	0.009
None	94(51.09%)	90(48.91%)		
Micro-Macro	66(67.35%)	32(32.65%)		
Prior_malignancy			0.57	0.45
No	181(58.96%)	126(41.04%)		
Yes	15(51.72%)	14(48.28%)		

**TABLE 3:** The chi-square test of the relation between risk score and clinical features in ICGC cohort.

Clinical feature	Risk score		c2	*p*
	High risk n(%)	Low risk n(%)		
Age			0.856	0.355
>65	49(35.25%)	90(64.75%)		
≤65	36(41.38%)	51(58.62%)		
Gender			0.023	0.881
MALE	53(34.42%)	101(65.58%)		
FEMALE	20(33.33%)	40(66.67%)		
Prior malignancy			0.201	0.654
Yes	12(41.38%)	17(58.62%)		
No	73(37.06%)	124(62.95%)		
Stage			10.132	0.001
I–II	41(29.50%)	98(70.50%)		
III–IV	44(50.57%)	43(49.43%)		

**TABLE 4:** The chi-square test of the relation between risk score and clinical features in GSE14520 cohort.

Clinical feature	Risk score		c2	*p*
	High risk n(%)	Low risk n(%)		
Age			1.162	0.281
>65	9(47.37%)	10(52.63%)		
≤65	119(60.10%)	79(39.90%)		
Gender			0.39	0.533
MALE	113(59.79%)	76(40.21%)		
FEMALE	15(53.57%)	13(46.43%)		
ALT			0.666	0.415
>50U/L	56(62.22%)	34(37.78%)		
≤50 U/L	72(56.69%)	55(43.31%)		
Main Tumor Size			6.126	0.013
>5 cm	54(70.13%)	23(29.87%)		
≤5 cm	74(52.86%)	66(47.14%)		
Multitumor			1.205	0.272
NO	97(57.06%)	73(42.94%)		
YES	31(65.96%)	16(34.04%)		
Stage TNM			13.417	<0.001
I–II	88(52.38%)	80(47.62%)		
III–IV	40(81.63%)	9(18.37%)		
Cirrhosis			11.241	<0.001
YES	125(60%)	75(40%)		
NO	3(17.65%)	14(82.35%)		
Stage BCLC				
0-A	88(53.33%)	77(46.67%)	9.095	0.003
B-C	40(76.92%)	12(23.08%)		
CLIP_Score				
≥2	33(68.75%)	15(31.25%)	2.429	0.119
<2	95(56.21%)	74(43.79%)		
AFP			1.103	0.294
>300 ng/ml	61(62.89%)	36(37.11%)		
≥300 ng/ml	67(55.83)	53(44.17%)		

### Relationship Between the Prognostic Model and the Tumor Microenvironment

**FIGURE 9:**
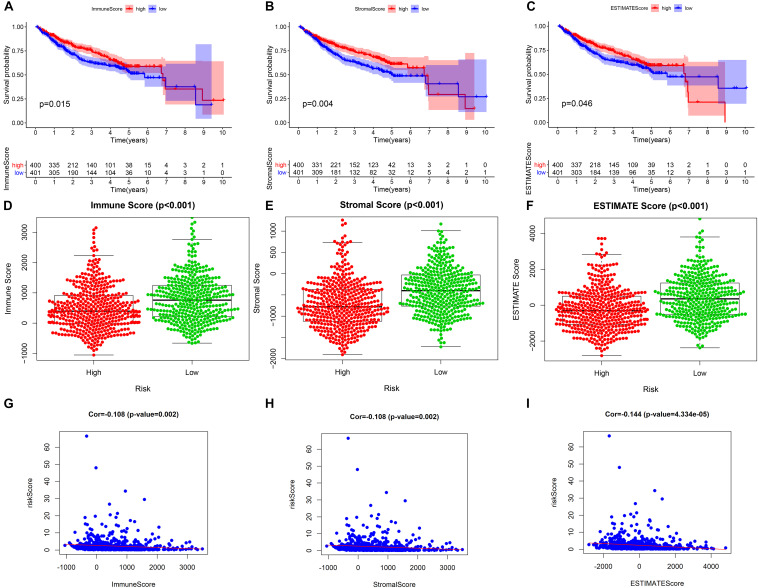
The relationship between the prognostic model and tumor microenvironment. **(A–C)** Kaplan–Meier survival analysis of patients in the whole cohort using the immune score, stromal score, and ESTIMATE score, respectively. **(D–F)** Comparison of the immune score, stromal score, and ESTIMATE score in the low- and high-risk groups, respectively. **(G–I)** Pearson correlation analysis between the risk score and immune score, stromal score, and ESTIMATE score, respectively.

Our study found that patients with higher tumor microenvironment scores (stromal score, immune score, and estimate score) had a better prognosis (Figures 9A–C), and the three kinds of tumor microenvironment scores were obviously higher in the low-risk group than in the high-risk group (*p* < 0.001) (Figures 9D–F), which further confirmed the obviously negative association of risk score with tumor microenvironment score by Pearson correlation analysis (Figures 9G–I). Therefore, we can assess the microenvironment of the tumor with the help of the risk score, which will provide a valid reference for the precise treatment of HCC.

### Gene Set Enrichment Analysis Between Different Risk Groups in Three Independent Cohorts

**FIGURE 10:**
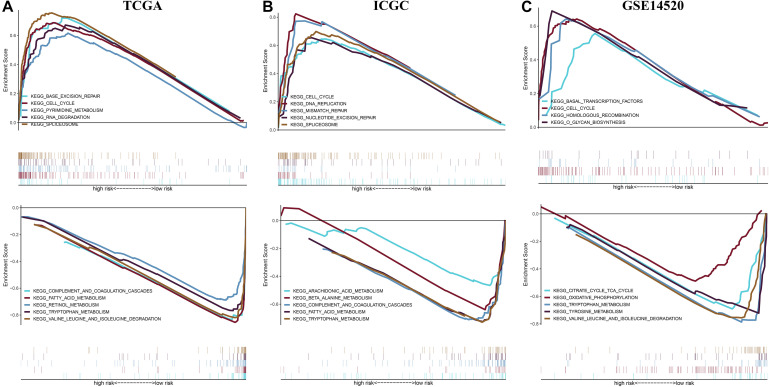
Gene set enrichment analysis between different risk groups in the **(A)** TCGA cohort, **(B)** ICGC cohort, and **(C)** GSE14520 cohort.

To reveal potential molecular mechanism for the prognostic model, we performed Gene Set Enrichment Analysis (GSEA) between different risk groups in three independent cohorts (Figure 10). The results showed that the high-risk group saw the enrichment of various tumorigenesis-related characteristics, including cell cycle, mismatch repair, homologous recombination, DNA replication, RNA degradation, etc. Most of the pathways that presented a significant enrichment in the low-risk group were related to metabolism. These results provide new clues for exploring the molecular mechanism of HCC in the future.

## Discussion

Hepatocellular carcinoma is the second leading cause of cancer-related death worldwide. During the past 10 years, HCC incidence has increased significantly (Yang et al., 2019). Surgical resection, liver transplantation, or radiofrequency ablation are only suitable for <30% of cases (Park et al., 2015). Sorafenib is the preferred treatment for patients with advanced HCC, but with the occurrence of high-frequency adverse events, there are no obvious improvements in the OS (Llovet et al., 2008). Recently, with the development of the immune microenvironment of the liver tissue, immunotherapy has become a new standard for the treatment of advanced HCC (Llovet et al., 2008).

Immunotherapy, represented by immune-checkpoint inhibitors (ICIs), has made a breakthrough in the clinical treatment of HCC (Hato et al., 2014). With the clinical application of ICIs, the exploration of biomarkers for cancer diagnosis, efficacy, and prognosis has become a hot topic in oncology immunotherapy research. With the development of DNA sequencing technology, the detection of the tumor mutation burden (TMB) has become a common experiment. The role of the TMB as a biomarker in predicting the efficacy of ICIs has been confirmed in a number of clinical studies (Ramalingam et al., 2018; Szustakowski et al., 2018; Vareki, 2018). However, research on the TMB in the prognosis of HCC is still lacking.

At present, how to define high TMB is still controversial worldwide (Chalmers et al., 2017). In this study, with the help of X-title software analysis, we found that when the TMB = 4.2 was used as the boundary between a high and low TMB, the difference in prognosis between the two groups was the greatest. HCC patients with high TMB had a poor prognosis, and these results are consistent with the conclusion of Cai et al. (2020). More interestingly, TMB = 4.2 was used as a cutoff, and the high- and low-TMB groups exhibited an obvious difference regarding immune cell infiltration. In particular, there was an obviously higher proportion of CD8 T lymphocyte infiltration in the high-TMB group than in the low-TMB group. Previous studies have shown that the effect of immunotherapy is positively correlated with the degree of tumor infiltration of CD8 T cells (Durgeau et al., 2018). Therefore, the TMB may further affect the efficacy of immunotherapy by changing the immune microenvironment of tumors, and it is necessary to explore the relationship between the TMB and immune genes.

Our LASSO Cox regression prognostic model included a total of 15 immune gene pairs, and each gene pair was associated with prognosis. To test the effectiveness of the prognostic model, we conducted three levels of validation. First, we split the training dataset, namely, the TCGA-ICGC joint cohort, and returned the patients to their original cohort for internal validation. Second, we regarded the GEO dataset as an external independent cohort to further verify the prediction effect of the model. Finally, we integrated all the samples into a whole cohort to test the prediction ability of the model again. Regardless of the level of validation, the prognosis model could accurately evaluate the prognosis of HCC. The uniform cutoff value was taken into account to divide all subjects into either high- or low-risk group, with the former presenting a weaker prognosis than the latter in each independent cohort, and the prognostic model served as an independent predictor in each independent cohort. Next, we explored the potential mechanism of the model through the tumor microenvironment (TME) score and gene set enrichment analysis. The patients with higher stromal, immune, and estimate scores showed good clinical outcomes, which corresponded to the higher level of the three scores in the low-risk group than in the high-risk group. In addition, GSEA revealed representative oncology features in the high-risk group. These results further confirmed the complex relationship among the TMB, TME, and tumor pathogenesis. According to the above results, we speculated that the change in the TMB may cause a series of cascade reactions, including the change in the immune gene expression levels, and the relative expression level of immune genes may affect the occurrence and development of tumors by disrupting the tumor microenvironment.

It is worth mentioning that we also compared the performance of our model with other reported models. For example, the AUC values of Liu et al. (2019) six-gene signature in predicting the 1-, 3-, and 5-year OS were 0.678, 0.643, and 0.633, respectively, and used the GSE14520 dataset for external validation, while the AUCs for our model predicting the OS at 1, 3, and 5 years were 0.677, 0.702, and 0.698, respectively, and were validated using the GSE14520. The AUC values of the (Li et al., 2019) six-gene signature in predicting the 1-, 3-, and 5-year OS were 0.681, 0.700, and 0.684, respectively, and used the ICGC dataset for the external validation, while the AUCs for our model predicting the OS at 1, 3, and 5 years were 0.906, 0.761, and 0.816, respectively, and were validated using the ICGC dataset. The above results demonstrated the better predictive performance of our model than that of the previous model.

The prognostic model involved 25 genes related to immunity. Studies have confirmed that some genes can affect HCC due to their occurrence, development, and immune regulation abilities. For example, Elise (Ramia et al., 2019) found that, in HCC cell lines, human leukocyte antigen (HLA) class II expression shortage was caused by the lack of the HLA class II transactivator (i.e., CIITA), and interferon-gamma treatment could not improve the situation. Lee et al. (2014) found that CD47(+) HCC cells secreted cathepsin S (CTSS) preferentially using the CTSS/protease-activated receptor 2 (PAR2) loop to regulate liver TICs. Calderaro et al. (2019) identified ESM1 as a reliable microenvironment immunohistochemical marker of macrotrabecular-massive HCC. Cho et al. (2016) revealed that the overexpression of FGR was significantly associated with a shorter time to tumor recurrence in HCC by means of integrative analysis. Chen et al. (2020) found an association between fibroblast growth factor receptor 4 (FGFR4) and the invasion and metastasis of HCC. Guo et al. (2016) demonstrated that elevated intracellular adhesion molecule 1 (ICAM-1) expression in HCC tissues was correlated with portal vein tumor thrombus development and poor clinical outcomes. Tang et al. (2015) found that a higher level of IKBKE in HCC tissues led to poorer HCC prognosis. Ni et al. (2019) demonstrated that the INS could be used to independently predict weak HCC prognosis, particularly for patients in the early stage of HCC. Carmo et al. (2016) speculated that PTX3 could be a risk factor for predicting the occurrence of HCC in chronic hepatitis C. Pinna et al. (2017) revealed that, in the signal transduction induced by liver tumor necrosis factor (TNF), the overexpression of TNFAIP3 inhibited c-JUN N-terminal kinase (JNK) phosphorylation and regulated the immune response given by non-parenchymal cells as well as HCC proliferation and apoptosis.

Although we tested the predictive effect of the prognostic model and explored its mechanism, our study is still a retrospective study. Therefore, it is necessary to carry out a multicenter prospective study before extending it to clinical application. Based on the study results, the relative expression possessed by a pair of immune genes associated with the TMB can significantly affect the prognosis of HCC, especially after the optimal threshold of the TMB has been determined. To our knowledge, similar reports are rare; hence, our future research directions are to reveal the underlying mechanism of this phenomenon through experiments.

## Conclusion

Our study explored the immune mechanism of HCC from the perspective of the TMB and established a reliable prognostic evaluation system, which may help to enrich the therapeutic targets of HCC.

## Data Availability Statement

The original contributions presented in the study are included in the article/Supplementary Material, further inquiries can be directed to the corresponding author.

## Author Contributions

JH designed the study, performed the data analyses and wrote the manuscript. LW and YZ contributed to the conception of the study. LW helped to perform the analysis with constructive discussions. All authors revised the manuscript and reviewed the final version of the manuscript.

## Conflict of Interest

The authors declare that the research was conducted in the absence of any commercial or financial relationships that could be construed as a potential conflict of interest.

## Footnotes

1 https://portal.gdc.cancer.gov/

2 https://icgc.org/

3 https://www.ncbi.nlm.nih.gov/geo/

4 https://www.immport.org/shared/genelists

## Supplementary Material

Supplementary Table 1 The clinical data of the three independent cohorts.

## Abbreviations

AFP: α-fetoprotein
DEIRGs: differential expressed immune-related genes
FDR: false discovery rate
GEO: Gene Expression Omnibus
GO: Gene Ontology
HCC: hepatocellular carcinoma
ICGC: International Cancer Genome Consortium
ICIs: immune-checkpoint inhibitors
IGPs: immune gene pairs
KEGG: Kyoto Encyclopedia of Genes and Genomes
LASSO: least absolute shrinkage and selection operator
OS: overall survival
RFS: recurrence-free survival
ROC: receiver operating characteristic
TCGA: The Cancer Genome Atlas
TIME: tumor immune microenvironment
TMB: tumor mutation burden
TME: tumor microenvironment.
