# Supplementary material for: A Prognostic Model of 15 Immune-Related Gene Pairs Associated With Tumor Mutation Burden for Hepatocellular Carcinoma
Source: Front Mol Biosci. 2020 Nov 13;7:581354. doi: 10.3389/fmolb.2020.581354 (PMC7691640; doi:10.3389/fmolb.2020.581354)
Supplement: Supplementary Table 1 — The clinical data of the three independent cohorts. [file Table_1.DOCX]

Table S1 The clinical data of the three independent cohorts

|  | TCGA(n=336) | ICGC(n=226) | GSE14520(n=239) |
| --- | --- | --- | --- |
| Survival status |  |  |  |
| alive | 222 | 185 | 143 |
| dead | 114 | 41 | 96 |
| Gender |  |  |  |
| female | 107 | 60 | 28 |
| male | 229 | 166 | 189 |
| Age |  |  |  |
| <=65 | 214 | 87 | 198 |
| >65 | 122 | 139 | 19 |
| Stage-TNM |  |  |  |
| I-II | 235 | 139 | 168 |
| III-IV | 82 | 87 | 49 |
| Prior malignancy |  |  |  |
| NO | 307 | 197 |  |
| YES | 29 | 29 |  |
| BMI |  |  |  |
| >25 | 149 |  |  |
| <=25 | 162 |  |  |
| With tumor |  |  |  |
| NO | 176 |  |  |
| YES | 145 |  |  |
| Histologic grade |  |  |  |
| G1-2 | 209 |  |  |
| G3-4 | 122 |  |  |
| Vascular invasion |  |  |  |
| NO | 184 |  |  |
| YES | 98 |  |  |
| New tumor event |  |  |  |
| YES | 164 |  |  |
| NO | 159 |  |  |
| Race |  |  |  |
| white | 164 |  |  |
| Asian&others | 162 |  |  |
| ALT |  |  |  |
| >50U/L |  |  | 90 |
| <=50U/L |  |  | 127 |
| Main Tumor Size |  |  |  |
| <=5cm |  |  | 140 |
| >5cm |  |  | 77 |
| Multitumor |  |  |  |
| NO |  |  | 170 |
| YES |  |  | 47 |
| Cirrhosis |  |  |  |
| NO |  |  | 17 |
| YES |  |  | 200 |
| Stage_BCLC |  |  |  |
| 0-A |  |  | 165 |
| B-C |  |  | 52 |
| CLIP_Score |  |  |  |
| <2 |  |  | 169 |
| >=2 |  |  | 48 |
| AFP |  |  |  |
| >300ng/ml |  |  | 97 |
| <=300ng/ml |  |  | 120 |
